# Exploring the influence of perceived classroom environment on learner autonomy in a Chinese EFL learning context

**DOI:** 10.3389/fpsyg.2022.1063473

**Published:** 2022-11-29

**Authors:** Shiyu Yang, Liyan Liu, Neil Hunt

**Affiliations:** ^1^Faculty of Education, Northeast Normal University, Changchun, China; ^2^School of Foreign Languages, Northeast Normal University, Changchun, China; ^3^Centre for English and Additional Languages, Lingnan University, Hong Kong, China

**Keywords:** learner autonomy, English classroom environment, high school students, EFL, Chinese context

## Abstract

Developing learner autonomy has been a critical task in English teaching that requires a clear understanding of the feature of classroom environment. This study aims to examine how senior high school students perceive classroom environment and learner autonomy, and how classroom environment exerts its influence on learner autonomy in Chinese EFL learning context. Participants (*N* = 565) from 15 classes located in northeast of China were selected to fill in an adapted version of What is Happening in This Class (WIHIC) and English Autonomous Learning Ability scale. Interview was conducted to confirm and illustrate the quantitative findings. The results revealed that senior high students had favorable perceptions of English classroom environment and learner autonomy. Grade differences existed in their perceptions. Moreover, we found that 53.7% of the variance in learner autonomy was accounted for by students’ perceptions of English classroom environment, which indicated that English classroom environment had significantly positive effects on learner autonomy. Specifically, task orientation, student involvement, teacher support and finding references were strong predictors to learner autonomy. The possible reasons for the findings were discussed and recommendations for future research were given.

## Introduction

In the field of 21st-century English language education, life-long learning and the learner-centered movement have created new challenges for students’ learning and teachers’ teaching, thus both language teachers and students need to reconsider their roles. Teachers are expected to become lifelong learners themselves and they play a crucial role in promoting their students’ autonomous learning ability (Ahmadianzadeh et al., 2020; Wang and Ryan, 2020; Ludwig and Tassinari, 2021; Ozer and Yukselir, 2021). As suggested by Holec (1981), the ultimate goal of education is to develop high level learner autonomy and help learners become autonomous learners. Learner autonomy, therefore, is one crucial factor in students’ learning and has increasingly attracted the attention of researchers and educators and becomes a central issue in the context of language learning and teaching in recent decades (Phan and Hamid, 2016; Lin and Reinders, 2019).

China published the New English Curriculum Standard in 2017, putting forward new requirements and expectations for English teaching and learning in senior high schools. There are four core competences that senior high students should possess and one of them is learning ability which refers to the awareness and ability that learners autonomously use and adjust English learning strategies, widen the English learning channel, and improve their learning effectiveness. The cultivation of learning ability can help learners improve their self-management, form good learning habits and learn English in an autonomous and effective way (Ministry of Education, 2018). It can be noted that autonomous learning, or in other words, learner autonomy, is given special attention in English language education reform in China.

Considerable research has stressed the critical role of learner autonomy in students’ learning. Cotterall (1995) demonstrates the effects of learner autonomy from the perspective of philosophy (maximizing learners’ rights to make choices), pedagogy (increasing efficiency and sense of security in learning) and practice (realizing proficiency). In addition, Benson (2007) comments that, learner autonomy closely interacts with a wide range of fields of language education, including motivation, individual difference, learning strategies, self-regulation as well as teacher development while Nakata (2010) believes that learner autonomy contributes to better outcomes in language acquisition. Learner autonomy also functions as a channel for learners’ individual and collaborative agency through which learners gradually develop language proficiency (Little, 2020).

Considering the importance of learner autonomy, different conditions have been put forward in the literature to promote the development of learner autonomy. Benson (2001) has highlighted that learner autonomy could develop if students are actively involved in the learning process and are given choices and flexibility of change. Kumaravadivelu (2011) has proposed different teaching strategies for teachers contributing to the promotion of learner autonomy. For instance, teachers can encourage learners to write diaries and journal entries, and enable them to develop critical thinking ability and interpretive capabilities. Teachers are also expected to play a role of facilitator to support students’ decision making, a resource provider to make him or herself a convenient resource when it is necessary, and a counselor to satisfy the ongoing need of students (Phan and Hamid, 2016; Ozer and Yukselir, 2021). Based on the literature, it is important to note that there is consensus on the great impact of classroom environment. Growing literature acknowledging that classroom environment is the key to developing learner autonomy has been published both in English (Ames, 1992; Eshel and Kohavi, 2003; Eneau and Develotte, 2012) and in Chinese (Sun, 2004; Fan, 2006; Li and Yin, 2010; Li, 2014; Li and Guo, 2015). As Paris and Paris (2001) point out, classroom environment can provide learner autonomy with social and material support by developing students’ cognitive strategies and motivation. Sun (2004) also argues that by giving learners supportive classroom environment, learners are able to maximize individual agency and autonomy in their learning.

Although much has been written about the key role of the classroom environment in improving learner autonomy theoretically, little empirical research has investigated the actual relationship between the two. The existing literature has yielded a dearth of empirical studies with regard to learner autonomy in senior high schools’ classroom environment in Chinese EFL context. It is necessary, therefore, to gather more critical and insightful evidence to authenticate how English classroom environment influences and shapes senior high students’ learner autonomy in the local context of China. Therefore, this study aims to examine senior high students’ perception of learner autonomy and the English classroom environment and explore how their perceptions of the English classroom environment influence learner autonomy. The following research questions are addressed in the study:

1. How do Chinese senior high school students perceive leaner autonomy?
2. How do Chinese senior high school students perceive their English classroom environment?
3. How does the perceived English classroom environment influence leaner autonomy in Chinese senior high schools?

## Literature review

### Conceptual framework of learner autonomy and classroom environment

Several attempts are made to define learner autonomy and the most remarkably robust and widely cited definition is Holec’s (1981) in which learner autonomy is regarded as the ability to take charge of one’s own learning and it was not inborn but developed either by formal learning or through natural means. Dickinson (1987, p.11) agrees with Holec and defines learner autonomy as “the situation in which the learner is totally responsible for all of the decisions concerned with his learning and the implementation of those decisions.” Little (1991) further adds a psychological dimension and points out that autonomy is a capacity for detachment, critical reflection, decision-making, and independent action, which indicates that the underlying psychological capacities are the core of learner autonomy.

Leaner autonomy is featured as complexity, multidimensionality, and variable manifestation (Benson, 2013), and there is a large body of literature highlights that leaner autonomy is a complex construct with different levels. For instance, Nunan (1997) proposes a five-level learner autonomy model of awareness, involvement, intervention, creation and transcendence. The basic level requires learners to be aware of learning goals, content and strategies followed by a deeper level in which they could be actively involved in learning and making choices. Learners would intervene in the learning process and adjust their learning goals, content and strategies in the third level. Whereas more autonomous learners develop learning content, create learning tasks, and go beyond the classroom, linking what they have learned to the outer world. In the field of language learning, Littlewood (1997) proposes a methodological framework for developing learner autonomy, which involves a three-stage model of language acquisition, learning strategies and individual development. Learners can use language independently to communicate with others in real situation in stage one. Stage two involves the ability of taking responsibility for learning and applying meaningful strategies and in the final stage, learner autonomy involves developing “greater generalized autonomy as individuals.” Similarly, Scharle and Szabo (2000) also describe three stages of becoming autonomous, which consist of raising awareness, changing students’ attitude and practicing new roles and habits, and transferring learners’ roles.

The understanding of classroom environment can track back to Lewin (1936) which firstly realizes the relationship between individuals and the environment, laying foundation for later research on learning environment. The classroom environment has been defined from different perspectives. For instance, from the physical perspective, Knirk (1979) defines classroom environment as a kind of learning site or place, which consists of classroom, library, playground, laboratory, school buildings and the learning areas at home. Fraser (1981) extends this understanding of classroom environment by arguing that it not only includes the classroom space, but also the relationship between teachers and students in a classroom, classroom quality, and classroom atmosphere. Later, Fraser (1986) further elaborates four components in the classroom environment including physical things, social interaction with others, the features of the members (teachers and students), and systems, concepts and values. The psychological aspects of the classroom environment have also been recognized. For example, Fan and Dong (2005) explain that classroom environment is the combination of physical, social and psychological factors where physical environment refers to the natural settings, teaching facilities and time–space environment, social environment includes the interaction between teachers and students, students and students, goal orientation, classroom rules and disciplines, and psychological environment concerns teachers’ and students’ personality traits, mental state and the psychological atmosphere in the classroom. This present study only discusses the social and psychological environment, focusing on learner-teacher interaction and learners’ psychological factors.

### Measurement of learner autonomy

With increasing research interest, the trajectory of learner autonomy research gradually shifts to classroom application and empirical investigation. Researchers develop a number of valid instruments to measure learner autonomy in different learning phases (Xu et al., 2004; Macaskill and Taylor, 2010; Oguz, 2013; Lin and Reinders, 2019), and investigate students’ and teachers’ beliefs, perceptions and readiness for learner autonomy in various educational contexts (Tanyeli and Kuter, 2013; Xiang and Wu, 2016; Bozkurt and Arslan, 2018; Lin and Reinders, 2019). There are a number of studies showing the close relationship between leaner autonomy and other key issues in learning and teaching, such as motivation (Huang and Liaw, 2007; Nakata, 2010; Calafato, 2020), self-regulation (Stefanou et al., 2013), learner achievement (Ghorbandordinejad and Nasab, 2016), teachers’ behavior and instruction (Grosmann and Wilde, 2020), language policy (Rivers, 2011; Phan and Hamid, 2016), and technology (Reinders and White, 2011; Lai, 2019; Stockwell and Reinders, 2019).

In the field of EFL learning and teaching, Liu and Li (2008) investigate 1,153 Chinese senior high school students’ learner autonomy and find that they have a negative perception of learner autonomy. Students in rural area have less favorable perceptions than those in urban schools while female students have better autonomy than male students. In the context of Chinese college English courses, Lin and Reinders (2019) examine undergraduates and teachers’ beliefs, practice and readiness for learner autonomy. The findings show that students and teachers are psychologically ready for autonomy, but not technically or behaviorally well-prepared. The Chinese English Curriculum Standard for Senior High School is the most important official plan on EFL teaching at upper secondary level in the country and its latest revision issued in 2017 puts special emphasis on the cultivation of learner autonomy. How do Chinese senior high students perceive their learner autonomy in the current stage? What problems do they encounter in developing their learner autonomy? This study tries to investigate senior high students’ perceptions of English learner autonomy and explore their difficulties in achieving autonomy.

### Measurement of classroom environment

Catering to different types of classroom environments, a variety of instruments are developed in recent decades. Firstly, The Learning Environment Inventory designed by Walberg and Anderson (1968) consists of 18 scales and 105 items whereas the Classroom Environment Scale includes nine dimensions (involvement, affiliation, teacher support, task orientation, competition, order, rule clarity, teacher control, and innovation) and 90 items (Trickett and Moos, 1973). My Class Inventory (Fisher and Fraser, 1981) is designed for science classroom environment and is a simplified version of the Learning Environment Inventory which contains five dimensions. What Is Happening In This Classroom (WIHIC; Fraser et al., 1996), which has been the most frequently used by researchers, contains 90 items and nine scales at first and is refined to 56 items with seven scales. Most of the above instruments are developed for math and science class and the existing instruments for English classroom are limited. Therefore, considering the characteristics of English classroom, Soebari and Aldridge (2015) adapts WIHIC by replacing the sub-scale “investigation” with “finding reference”.

Classroom environment has been widely researched in different disciplines such as Math (Aluri and Fraser, 2019), Science (Fraser and Aldridge, 2010), and Biology (Zeidan, 2010). It has also been studied in terms of EFL learning in different context which focuses on instruments development, learners and teachers’ perceptions of actual classroom environment (Goksu, 2015), and how English classroom environment exerts its influence on learners’ achievement (Gedamu and Siyawik, 2015), attitude (Lim and Fraser, 2018), and engagement (Hoi, 2022). For instance, Goksu (2015) examines how 166 Turkish high school students perceive their English classroom environment and the findings reveal that participants have positive perceptions and there are no grade and gender differences in their perceptions. In addition, Gedamu and Siyawik (2015) explore the association between secondary school students’ perceived English classroom environment and English learning achievements in Ethiopia. The results indicate that task orientation, student involvement, and teacher support positively correlate with their English language test score. Lim and Fraser (2018) conduct their research in Singaporean primary school and find that English classroom environment seems to have positive correlation with attitude to English learning and academic efficacy. Furthermore, research conducted in China (Liu and Liu, 2012) which focuses on the correlation between senior high school students’ perceptions of English classroom environment and academic outcome shows that students perceive their English classroom environment positively and female students tend to have better perceptions than male students do. The study also indicates that task orientation contributes to learner outcome.

### Developing learner autonomy in English classroom environment

Different research has increasingly stressed the interplay between the classroom environment and learner autonomy. According to Willis (2011), classroom learning is mutual work between students and teachers and learner autonomy is usually developed in classroom setting. Teachers should take on the responsibilities to create positive classroom environment in which students could recognize their own role and be more autonomous in learning. Furthermore, Li and Yin (2010) investigate 1,995 middle school students in Hong Kong and find that teacher support and involvement contribute to learner motivation and the use of self-regulated learning strategies. Both teacher-centered and learner-centered classroom environments are helpful in cultivating learner autonomy. The above findings are in line with Sungur and Güngören’s (2009) research which reaches the conclusion that students’ perceived science classroom environment positively associates with learner regulation and science achievement. In another research on undergraduate students in a private university finds that problem-based and project-based classroom environments support students’ engagement in self-regulation learning (Stefanou et al., 2013). It can be seen from the previous research that classroom environment plays a facilitated role in enhancing learners’ engagement and responsibility in learning.

In the context of EFL learning, some empirical studies in China shed some lights on the impact of the English classroom environment on learner autonomy. Li (2014), for example, explores the correlation between non-English major undergraduates’ perception of English classroom environment and their learner autonomy. The findings indicate that they perceive classroom environment favorably and their autonomous learning ability is at a moderate level. The classroom environment’s influence on learner autonomy is greater than learner autonomy’s effects on classroom environment. Students involvement, students responsibility, task orientation and teacher support are the most significant predictors of learner autonomy. Similarly, the investigation of Li and Guo (2015) suggests that classroom environment of non-English major undergraduates contributes to their learner autonomy. Specifically, both teaching (teacher leadership, teaching innovation, and teacher support) and learning (partner relationships, class involvement, and task orientation) in English classroom environment favorably correlate with learner autonomy.

Reviews of research clearly indicate that western scholars noticed the critical role of classroom environment on the development of learner autonomy (Ames, 1992; Willis, 2011; Doğan and Mirici, 2017). However, little empirical research was conducted to investigate the actual relationship between them and to what extent the former may contribute to the latter in EFL learning. A number of Chinese scholars tries to fill this gap by investigating the correlation between English classroom environment and learner autonomy at tertiary level (Li, 2014; Li and Guo, 2015). However, how English classroom environment influences learner autonomy at secondary level is under-researched. It is, therefore, necessary to understand what elements in English classroom environment make it more or less likely that learners will develop leaner autonomy in secondary school context. The present study attempts to address this gap in the literature by exploring senior high school students’ perceptions of English classroom environment and learner autonomy and how the perceived classroom environment influences learner autonomy.

## Materials and methods

### Research design

A mixed method design was carried out in the study. Two questionnaires were employed to examine students’ perception of English classroom environment and learner autonomy. Interviews with students were conducted to elicit in-depth data about their feelings and opinions related to their English classroom and their own English learning habits and methods.

### Participants

The sample comprised a total of 565 students from two senior high schools, in Jilin and Liaoning province located in northeast China. The participants were from 11 classes each of which involved 45–51 students and they all learned English as a Foreign Language. With regard to age, it ranged from 15 to 17. There were 301 students at grade 10 (53.3%) and 264 students at grade 11 (46.7%). Grade 12 students were excluded from the present research for the reason that they were preparing for the college entrance examination, and it was hard to find enough time to complete the questionnaire and conduct interviews. The distribution of gender was relatively balanced in the study which consisted of 281 male students (49.7%) and 284 female students (50.3%).

### Data instruments

#### English autonomous learning ability

The questionnaire English Autonomous Learning Ability (Xu et al., 2004) was adopted in the present research to quantitatively measure students’ perception of learner autonomy. It is composed of five sub-scales and 32 items. Each of the first three sub-scales contains five items, in which learners self-report their perceptions of knowing the teaching purpose and requirements, setting learning goals and making plans, and using different learning strategies, respectively. The fourth sub-scale consists of seven items assessing how learners monitor their use of strategies. The fifth sub-scale includes 10 items that measures how learners monitor and assess their English learning process. This questionnaire uses Likert scales with five categories of points (1—Almost Never, 2—Seldom, 3—Sometimes, 4—Often, and 5—Almost Always) to solicit students’ responses to learner autonomy. The Cronbach’s alpha was 0.94, which suggested a high reliability. Regarding the validity, the questionnaire was proved to be valid (X2/df = 2.80, RMSEA = 0.05, IFI = 0.92, CFI = 0.92, TLI = 0.90).

#### What is happening in this class

What is Happening in This Class (WIHIC) is one of the most frequently used instruments to measure the classroom environment and was developed by Fraser et al. (1996). Seven scales and 56 items were included in WIHIC, namely students’ cohesiveness, teacher support, involvement, investigation, task orientation, cooperation and equity. Soebari and Aldridge (2015) adapted WIHIC to measure students’ perceptions of English classroom environment by replacing the investigation sub-scale with finding reference. The reason it that investigation is most frequently used in science classes while finding information with the help of dictionary or reference books is more commonly used in English classes. There are two reasons for choosing the revised version of WIHIC. First, the adapted version considers the features of English classroom, thus it is more suitable for the present study than the original version of WIHIC. In addition, it is widely used and has proved to be more valid and reliable. Accordingly, an acceptable Cronbach’s alpha of 0.95 was found and a high validity was reported for the revised WIHIC in the present study (X2/df = 2.51, RMSEA = 0.05, IFI = 0.90, CEI = 0.91, TLI = 0.90).

### Data collection

As previously mentioned, both quantitative methods and qualitative methods were used to collect data from the senior high school students *via* questionnaire and interview which allowed a more in-depth understanding of students’ perceived learner autonomy and English learning environment through triangulation. First, revised WIHICH and English Autonomous Learning Ability questionnaires were distributed to 600 students anonymously in 2019. Then, based on different gender and grades, eight students were selected purposefully for an interview that lasted for an average of 30 min. There were two female students and two male students from grade 10 and grade 11, respectively. Since the participants would better express their opinions in mother tongue, Mandarin was used in all interviews. The interviews were recorded and transcribed into written text to help illustrate the research findings.

### Data analysis

For the quantitative data, the scores obtained from two questionnaires were analyzed in SPSS 20.0. Specifically, descriptive analysis was applied to evaluate students’ general perceptions of English classroom environment and learner autonomy. Besides, an independent-sample *t*-test was used to explore whether there were gender and grade level differences in their perceptions. To investigate the relationship among the seven sub-scales of WIHIC and five sub-scales of English Autonomous Learning Ability, a two-tailed Pearson Product–Moment Correlation was conducted at a significant level of 0.05. Multiple regression analysis was carried out to examine how students’ perceptions of English classroom environment influences their perceptions of learner autonomy and we used beta weights of multiple regressions to see each predictor variable’s relative contributions. With regard to the qualitative data, the interviews were classified into different sections that are parallel with the sub-scales in the two questionnaires. Finally, students’ citations in the interview were used in presenting the qualitative data results and they were given number as G10F1 or G11M1 based on their grade and gender.

## Results

### Students’ perceptions of learner autonomy

As shown in Table 1, the mean score of learner autonomy scale is 3.43 (SD = 0.63), which indicates that senior high students have a positive attitude toward learner autonomy in English learning than average level. Generally speaking, they are ready to take responsibilities for their English learning. Specifically, knowing teaching purpose and requirements scale has the highest mean score (M = 3.65, SD = 0.74) and using different learning strategies has the lowest mean score (M = 3.31, SD = 0.78). The qualitative data also shows that senior high students know what they are expected and what they should do in English learning (see extract 1), but they lack enough knowledge of English learning strategies in listening, speaking, reading, and writing (see extract 2).

Extract 1 *I am not clear about teaching purposes because my teacher hardly explains that to us. Most of the time, I just complete tasks and answer questions. I know the requirements for English learning very well. Our teacher made all the rules and requirements clear when we entered high school. For example, we should stay focused, take notes if necessary, keep writing individual diary, preview the reading passage in each unit, and review on time (G10F1).*

Extract 2 *I usually do a lot of exercises and then I can find the rules. Doing exercise is helpful to improve my English. As for reading and writing, I think it’s necessary to memorize more vocabulary, which makes reading and writing easier…In my opinion, English writing is similar to Chinese writing. I get used to organizing the structure and sentences in Chinese and then translating them into English (G10M2).*

**Table 1:** Descriptive analysis of learner autonomy.

Scales	K	S	U	M	MaA	Total
M	3.65	3.52	3.31	3.35	3.44	3.43
SD	0.74	0.81	0.78	0.64	0.65	0.63

K, Knowing teaching purpose and requirements; S, Setting learning goals and making plans; U, Using different learning strategies; M, Monitoring the use of strategies; MaA, Monitoring and assessing English learning process.

The findings presented in Table 2 demonstrate that no statistically significant difference is found in gender for most of the dimensions of learner autonomy. However, the analysis also shows that female students outperform male students on the sub-scale of knowing teaching purpose and requirements (*t* = −2.09, *p* < 0.05). Regarding the grade difference, the analysis indicates that there is a statistically significant difference for most of the sub-scales. Grade 11 students score significantly higher than grade 10 students on the sub-scales of knowing teaching purpose and requirements (*t* = −2.85, *p* < 0.01), using different learning strategies (*t* = −8.05, *p* < 0.001), monitoring the use of strategies (*t* = −6.32, *p* < 0.001), and monitoring and assessing English learning process (*t* = −5.27, *p* < 0.001). Considering the qualitative data, it also shows that grade 11 students employ more diverse learning methods (see extract 3) while grade 10 students lack the knowledge of different learning strategies on listening, speaking, reading and writing, and they rely heavily on memorization and doing exercise (see extract 4).

Extract 3 *I practice English by taking part in some activities in our school, such as English Corner and drama performance. I also use some applications namely English Dubbing and Scallop Vocabulary to practice my oral English and enlarge my vocabulary (G11M2).*

Extract 4 *I practice my English by doing exercise, by which I can find the rules and get good grades in the exams…I also spend lots of time remembering vocabulary and paragraphs in the reading passages (G10M1).*

**Table 2:** The results of gender and grade differences in learner autonomy.

Scales	M		SD		*t*	*p*	M		SD		*t*	*p*
	M	F	M	F			G10	G11	G10	G11		
K	3.59	3.72	0.82	0.65	−2.09	0.03^*^	3.57	3.75	0.79	0.67	−2.85	0.004^**^
S	3.47	3.57	0.86	0.77	−1.35	0.17	3.50	3.55	0.87	0.74	−0.82	0.40
U	3.32	3.30	0.80	0.75	0.30	0.76	3.07	3.57	0.79	0.68	−8.05	0.000^***^
M	3.33	3.37	0.68	0.60	−0.56	0.57	3.19	3.53	0.63	0.61	−6.32	0.000^***^
MaA	3.43	3.45	0.66	0.63	−0.31	0.75	3.31	3.59	0.68	0.57	−5.27	0.000^***^

M, male; F, female; G10, grade 10 students; G11, grade 11 students.

* *p* < 0.05.

** *p* < 0.01.

*** *p* < 0.001.

### Students’ perceptions of English classroom environment

Descriptive statistics for students’ perceptions of English classroom environment are presented in Table 3. It can be seen that students have positive classroom environment perceptions in EFL classes (M = 3.70, SD = 0.55). Among the seven sub-scales, student cohesiveness is scored highest by students (M = 4.08, SD = 0.60), and finding reference obtains the lowest mean (M = 3.34, SD = 0.80). It indicates that sample senior high school students have a close relationship with their peers and they tend to help and support each other in English class. On this issue, one participant G10F2 said:

Extract 5 *My classmates often help me learn English. If I have trouble in some grammar rules, they are willing to explain that to me. They do not laugh at me even if I ask a very simple question or make a silly mistake. Sometimes I cannot follow the teacher’s speed in class, my classmates lend their notes to me (G10F2).*

**Table 3:** Descriptive analysis of English classroom environment.

Scales	SC	TS	SI	FR	TO	C	E	Total
M	4.08	3.72	3.38	3.34	3.71	3.62	3.97	3.70
SD	0.60	0.71	0.67	0.80	0.67	0.71	0.70	0.55

SC, student cohesiveness; TS, teacher support; SI, student involvement; FR, finding reference; TO, task orientation; C, cooperation; E, equity.

Table 4 presents the analysis of gender and grade differences on the sub-scales of the WIHIC. The results of independent t-test show that of all seven sub-scales examined, only student involvement is found to have significant gender difference (*t* = 2.91, *p* < 0.05). Male students outperform female students regarding their perceptions of student involvement, and they tend to be more active and better involved in class than female students do. The following extract confirms this finding.

Extract 6 *Our English teacher always encourages us to be active and brave in English class. However, I usually participate in activities and express myself only if I am quite confident about the topic and know the exact answers. Before expressing my ideas, I must think it over and organize my words ahead of time. I am a little bit worried about whether my answers are correct (G11F1).*

**Table 4:** The results of gender and grade differences in English classroom environment.

Scales	M		SD		*t*	*p*	M		SD		*t*	*p*
	M	F	M	F			G10	G11	G10	G11		
SC	4.09	4.08	0.58	0.62	0.88	0.93	4.08	4.09	0.61	0.59	−0.19	0.844
TS	3.77	3.67	0.70	0.71	1.79	0.07	3.56	3.90	0.79	0.56	−5.91	0.000^***^
SI	3.46	3.30	0.67	0.67	2.91	0.004^**^	3.26	3.51	0.71	0.60	−4.54	0.000^***^
FR	3.31	3.36	0.86	0.73	−0.72	0.47	3.23	3.45	0.84	0.73	−3.32	0.001^**^
TO	3.66	3.7	0.72	0.61	−1.75	0.08	3.68	3.74	0.68	0.65	−1.13	0.258
C	3.61	3.63	0.71	0.71	−0.39	0.69	3.58	3.67	0.77	0.63	−1.46	0.144
E	3.95	3.99	0.69	0.70	−0.65	0.51	3.91	4.03	0.76	0.61	−2.02	0.043^*^

M, male; F, female; G10, grade 10 students; G11, grade 11 students.

* *p* < 0.05.

** *p* < 0.01.

*** *p* < 0.001.

As to the grade differences, grade 11 students’ perceptions of teacher support (*t* = −5.91, *p* < 0.001), student involvement (*t* = −4.54, *p* < 0.001), finding reference (*t* = −3.32, *p* < 0.01), and equity (*t* = −2.02, *p* < 0.05) are significantly higher than grade 10 students.

### Influence of English classroom environment on learner autonomy

The second research question aims to investigate the effects of students’ perceived English classroom environment on learner autonomy. Table 5 presents the results of multiple regression analysis between the predictor variable and dependent variables. Accordingly, the combination of English classroom environment variables significantly predict learner autonomy (*F* = 164.24, *p* < 0.05). Adjusted R^2^ is 0.537, which indicates that 53.7% of the variance in learner autonomy is accounted for by students’ perceptions of English classroom environment. Additionally, as indicated in Table 5, task orientation is the most significant predictor of students’ perceptions of learner autonomy (*β* = 0.378; *t* = 9.689, *p* < 0.001). Student involvement (*β* = 0.258; *t* = 5.741, *p* < 0.001), teacher support (*β* = 0.123; *t* = 2.906, *p* < 0.01), and finding reference (*β* = 0.125; *t* = 3.540, *p* < 0.01) also positively contribute to learner autonomy.

**Table 5:** Results of multiple regression analysis between English classroom environment and learner autonomy.

Dependent variable	Predictors	Beta	R^2^	R^2^ adj	F	*t*
Learner autonomy	Task orientation	0.378	0.540	0.537	164.24	9.689^***^
	Student involvement	0.258				5.741^***^
	Finding reference	0.125				3.540^**^
	Teacher support	0.123				2.906^**^

** *p* < 0.01.

*** *p* < 0.001.

## Discussion

In this study, we aim to examine senior high students’ perceived learner autonomy, classroom environment, and the interplay between them in Chinese EFL learning context. The first goal is to investigate their perceptions of learner autonomy and the results display that they perceive it positively, which indicates that they can take some responsibilities for English learning. This finding contradicts the previous study reporting that Chinese senior high students have a poor perception of English learner autonomy, and specifically, they cannot use and monitor strategies effectively in reading and writing (Liu and Li, 2008; Wang and Hao, 2010). This can be partly accounted for by the Core Competency Reform in basic education in China which began with the new curriculum standard issued in 2017. The shift of control from teachers to students is gradually recognized and English teachers are becoming more leaner-centered and humanistic in language teaching. For instance, they prepare learning materials with guided questions, asking students to preview before the class, discuss with peers in class, and solve the problems they encountered (Meng, 2018). Meaningful, individualized and project-based homework is given to students, which emphasizes students own enquiry and activities. This way of teaching highlights the significance of students’ freedom and responsibilities in English learning and helps them control and regulate the learning process autonomously. Therefore, with the progress of Core Competency Reform, Chinese senior high school students in the present study have higher level of English learner autonomy compared with students in last decade.

Furthermore, we also find that grade 11 students report a higher level of perception than grade 10 students in most sub-scales of learner autonomy, which shows that learner autonomy increases with progression through learning. While this finding is inconsistent with the previous studies (Lee et al., 2009; Xiong et al., 2017) which draw the conclusion that lower graders have better perceptions of learner autonomy than higher graders, it is conceivable in the context of this study. As is noted in the interview, grade 10 students experience frustration and encounter some difficulties at the beginning of high school study. It is difficult for them to make decision and regulate learning process independently in a transition period. However, due to the increase of learning experience, grade 11 students have clearer awareness of their own strengths and weaknesses, and they are more familiar with English learning process. Thus they can choose a variety of appropriate learning methods flexibly according to their own situation and manage the learning process independently. With regard to the gender differences on students’ learner autonomy, the result in the present research indicates that there is no significant gender differences, which is congruent with the findings of Lee et al. (2009). Both male and female students are responsible for goal-making, planning, initiating and evaluating their English learning strategies. However, some studies report a gender difference on students’ learner autonomy and find that girls view themselves more autonomous than boys (Liu and Li, 2008; Wang and Hao, 2010).

The second aim of this study is to examine Chinese high school students’ perceptions of English classroom environment and the findings indicate that they have a positive attitude toward that. This result is in line with previous research suggesting favorable classroom environment in EFL context (Liu and Liu, 2010; Goksu, 2015; Lim and Fraser, 2018). High school students feel a sense of academic and emotional support from their teacher and peers and they are actively involved in English class. As indicated by Pishghadam et al. (2020a), if students gain emotional support and have high level of emotioncy (a blend of individuals’ emotions and senses), they tend to show more willingness in involvement. High school students also perceive their English classroom task-oriented, equal and cooperative, which might be explicated in terms of the idea of activity-based and competency-oriented English teaching which have been highlighted since the Core Competency Reform stared (Ministry of Education, 2018). Contextualized, topic-specific and problem-solving activities are presented in class. They are relevant to students’ interests and goals as well as connected to students’ lives (Li and Tian, 2021). Activity-based English classroom can enhance students’ motivation and participation, and create opportunities for learner interaction and collaboration, thus eliciting positive cognitive response from students.

The finding regarding the gender differences shows that no statistically significant gender difference exists in the perceptions of English classroom environment. It is in parallel with the previous study conducted by Goksu (2015) in Turkish EFL context. Although some studies find that female students better perceive classroom environment than male students in many aspects, such as task-orientation, cooperation (Liu and Liu, 2012; Tshewang et al., 2017; Lim and Fraser, 2018) and teacher support (Gherasim et al., 2013), our research indicates that male students outperform female students in student involvement scale, suggesting that they are more engaged in English classroom. This might be explained with regard to the social-cultural factors that many Chinese girl students, relative to the male counterparts, are more cautious and conservative in nature. They tend to engage in the classroom activities unless they are fully well-prepared and confident about themselves. In addition, we do find significant grade differences in most dimensions of classroom environment. Compared with grade 10 students, grade 11 students tend to have more favorable perceptions of teacher support, student involvement, finding reference and equity. One possible reason is that grade 11 students are gradually familiar with their English class and teacher’s teaching style, and benefit from teacher’s scaffolding. For another, facing the pressure of University Entrance Examination, they become more responsible for themselves and try to find resources to deal with problems in their English learning.

The last purpose of the research is to investigate the influence of classroom environment on learner autonomy in Chinese EFL context. Overall, results suggest that senior high school students’ perceived classroom environment exerts positive influence on their learner autonomy. It could be supported by Li’s research (2014) conducted at tertiary level which reports the significant role of classroom environment in the development of learner autonomy. He also argues that teacher support, task orientation, student involvement, and student responsibility have significant effects on students’ abilities to make learning plans, use and monitor strategies, and assess English learning process.

Specifically, our results identify four vitally important variables that have positive effects on learner autonomy, namely task orientation, student involvement, teacher support, and finding reference. Firstly, task orientation refers to the extent to which students attach importance to complete planned tasks, focus on subject matter and coordinate class activities (Lai et al., 2015), and it is the strongest predictor of Chinese senior high school students’ learner autonomy. The result confirms the findings of Li and Guo (2015) who report that the more task-oriented university students are, the more independent they will be. One possible reason is that classroom characterized with high task orientation develop students’ habits of making preparations before the class, keeping focus on learning goals in class, and employing different methods to complete goals and tasks. Accordingly, it leads to students gradually assume responsibilities in learning and practicing English. Second, the significant effect of student involvement on learner autonomy is congruent with considerable previous studies (Patrick et al., 2007; Li and Guo, 2015; Wang and Ryan, 2020). As Holec (1981) stated, centering the focus on learning rather than teaching is the core concept of learner autonomy. By actively engaging in classroom activities, such as group discussion, individual study, group presentation, and peer feedback, students are placed at the centering of learning, taking control and exercising agency. Learner autonomy is fostered when students have opportunities to makes choices over their learning behavior and manage learning process actively. Third, teacher support contributes to students’ autonomous learning, and it echoes the findings of the research of Lee et al. (2009) which find that teacher support is the most influential factor of Hong Kong students’ self-regulated learning and strategy use. As is mentioned in previous research (Patrick et al., 2007), if students view their English teacher as being academically and emotionally supportive, they are more likely to develop clear orientation to English learning, fully engage in academic activities with self-regulated learning strategies, and promote their intrinsic motivation. Hence, their willingness to engage language learning and autonomous learning ability is facilitated. Fourth, finding reference refers to the extent to which English classroom emphasizes the skills and process of solving problems by using references (such as dictionary, reference book, technology tools, etc.) to find information and resources by students themselves (Soebari and Aldridge, 2015). Classroom that encourages finding references develops a better awareness and behavior for taking more responsibilities in solving problems in language learning. Students are equipped with the ability to learn English with various channels and reference on their own even if they are remote from classroom, thereby promoting leaner autonomy.

## Limitations and recommendations for future research

The present research provides an empirical validation for senior high school students’ perception of leaner autonomy and classroom environment in Chinese EFL context after the Core Competency Reform. It expands our knowledge of learner autonomy to its association with the English classroom environment and documents how English classroom environment positively influences students’ autonomous learning ability. Moreover, it casts lights on the directions for improving learner autonomy by cultivating task-oriented, teacher-supported, engaging and independent classroom environments. Despite the above contributions, some limitations of this study also need to be noted. First, this study only includes participants from northeast of China, which may render the generalization of the study finds. Larger sample size and equal distribution of participants are needed in future research to verify the findings of this research. In addition, although we confirm classroom environment’s positive effects on learner autonomy, it has to admit that there are some other possible influencing variables that are overlooked in the present research. For instance, some socio-cultural variables and psychological variables may influence students’ perceptions of classroom environment and learner autonomy, which should be taken into consideration for future studies when conducting research in various context. Socioculturally, culture affects individuals’ thoughts, behavior and language learning. The relationship between language and culture is called “cultuling” by Pishghadam et al. (2020b) and they also pointed that cultural memes, defined as cultural genes and units of cultural transmission, shape individuals’ beliefs and behavior (Pishghadam et al., 2020b). In other words, students with different cultural backgrounds and cultural memes may have different language learning behavior in their English classroom and diverse perceptions of their classroom environment. Psychologically, learners’ emotional involvement can impact language learning (Akbari and Pishghadam, 2022). For instance, emotioncy, a newly-developed concept that emphasizes the intertwined state between emotions and senses (Pishghadam et al., 2015), also impacts individuals’ cognition, thoughts, behavior and interaction (Pishghadam et al., 2020b). In language learning, learners gain high level of emotioncy when teachers have sufficient emo-sensory intelligence and provide multiple senses in classroom environment, leading to learners’ active involvement and willingness to communicate and interact with others (Pishghadam et al., 2015, 2019, 2022; Akbari and Pishghadam, 2022). It is highly recommended to include these variables in future research, such as cultural memes, emotioncy, and motivation, which may play a mediating or moderating role between classroom environment and learner autonomy.

## Data availability statement

The original contributions presented in the study are included in the article/Supplementary material, further inquiries can be directed to the corresponding author.

## Ethics statement

The studies involving human participants were reviewed and approved by Northeast Normal University. The patients/participants provided their written informed consent to participate in this study.

## Author contributions

All authors listed have made a substantial, direct, and intellectual contribution to the work and approved it for publication.

## Funding

This work was supported by National Social Foundation of China: The career trajectory of female primary school teachers and its influencing factors (Grant number: BHA180155).

## Conflict of interest

The authors declare that the research was conducted in the absence of any commercial or financial relationships that could be construed as a potential conflict of interest.

## Publisher’s note

All claims expressed in this article are solely those of the authors and do not necessarily represent those of their affiliated organizations, or those of the publisher, the editors and the reviewers. Any product that may be evaluated in this article, or claim that may be made by its manufacturer, is not guaranteed or endorsed by the publisher.
